# Multiscale Road Extraction in Remote Sensing Images

**DOI:** 10.1155/2019/2373798

**Published:** 2019-07-10

**Authors:** Aziguli Wulamu, Zuxian Shi, Dezheng Zhang, Zheyu He

**Affiliations:** ^1^School of Computer and Communication Engineering, University of Science and Technology Beijing (USTB), No. 30 Xueyuan Road, Haidian District, Beijing 100083, China; ^2^Beijing Key Laboratory of Knowledge Engineering for Materials Science, Beijing, China

## Abstract

Recent advances in convolutional neural networks (CNNs) have shown impressive results in semantic segmentation. Among the successful CNN-based methods, U-Net has achieved exciting performance. In this paper, we proposed a novel network architecture based on U-Net and atrous spatial pyramid pooling (ASPP) to deal with the road extraction task in the remote sensing field. On the one hand, U-Net structure can effectively extract valuable features; on the other hand, ASPP is able to utilize multiscale context information in remote sensing images. Compared to the baseline, this proposed model has improved the pixelwise mean Intersection over Union (mIoU) of 3 points. Experimental results show that the proposed network architecture can deal with different types of road surface extraction tasks under various terrains in Yinchuan city, solve the road connectivity problem to some extent, and has certain tolerance to shadows and occlusion.

## 1. Introduction

Road extraction in remote sensing images is a very important and challenging task. It is of great significance for map composition, effective precision agriculture, urban planning, etc. At present, road extraction generally uses manual visual interpretation, which is very time consuming and might produce different results due to the interpreter's professionalism. In the acquisition of remote sensing images, there is a range of inevitable problems, such as terrain differences, shadows, and occlusions. These problems will pose certain challenges to road extraction. Meanwhile, it is difficult to develop a general road extraction architecture because the definitions of roads and the standards for road construction are varied in different countries.

In recent years, with the development of CNN [1], CNN has played an increasingly important role in delivering computer vision and addressing natural language processing problems. Semantic segmentation is an important task based on CNN. It is mainly used in computer vision applications such as automatic driving, remote sensing image interpretation, and medical image processing. This task has undergone significant progress, with excellent network architecture such as FCN [2] and U-Net [3]. FCN is the pioneering work of semantic segmentation with CNN. U-Net was first proposed in the field of medical image processing and then widely used in small sample semantic segmentation. It can not only consider the use of context information but also accurately locate semantic segmentation objects. However, simple extracting multiscale context information in remote sensing images is inadequate.

In this paper, our goal is to solve the road extraction in three urban areas of Yinchuan city, with a total area of 2,045 square kilometers. These areas have complex terrain, including mountains, alluvial plains, deserts, cultivated land, urban areas, photovoltaic power plants, and woodlands. The shape of roads in different terrains is also different. To this end, we propose a network structure based on encoder-decoder and atrous spatial pyramid pooling [4]. Meanwhile, a combination of multiple loss functions is adopted as the final loss function. Experiments show that this proposed method attains performance of 82.3% on the Yinchuan city test set without any postprocessing. Therefore, this method can better solve the road extraction problem in the region.

The rest of the paper is arranged as follows. In Section 2, the related work with road extraction is described. In Section 3, this method is elaborated by splitting different modules.  Section 4 presents the detailed experimental data, evaluation criteria, and experimental results. Finally, the conclusion is given in Section 5.

## 2. Related Work

### 2.1. Convolutional Neural Networks

CNN is a neural network designed to process data with a similar grid structure. It is stacked in order by convolutional layer, pooling layer, activation function layer, and a fully connected layer. Inputting an image, CNN can output the score value of the classification corresponding to the image. CNN was the first neural network to solve commercial applications. As early as the 1990s, LeNet-5 [5] was successfully applied to digits recognition, which was deployed commercially and read millions of checks per day. In 2012, AlexNet [6] was the winner of the ILSVRC [7]. AlexNet solves the problem of image classification and forged a new landscape in computer vision. Then, the top competitors proposed varieties of CNN architectures, such as GoogleNet [8], ResNet [9], DenseNet [10], and others [11]. These network architectures can extract feature maps of images well and lay a solid foundation for semantic segmentation. In particular, ResNet solves the problem of “increasing error rate as the network deepens.” Our network architecture uses ResNet34 as feature extractor.

### 2.2. Semantic Segmentation

Semantic segmentation is the classification of each pixel in an image. It evolved from image classification. FCN is the pioneer work of semantic segmentation with CNN. It converts the fully connected layer in the traditional CNN into a convolutional layer and then upsamples to restore the resolution of the original image. U-Net uses skip connections and encoder-decoder architecture for more accurate results. SegNet [12] solves the problem of location information loss by using pooling with the indices of max locations. RefineNet [13] exploits features at multiple levels of abstraction for high-resolution segmentation and proposes “chained residual pooling” which is able to capture background context from a large image region. LinkNet [14] can quickly perform semantic segmentation while guaranteeing accuracy. PSPNet [15] uses spatial pyramid pooling techniques to obtain multilevel semantic features. Based on the U-Net architecture, there are many excellent semantic segmentation networks, such as W-net [16], U-Net++ [17], and Multispectral U-NET [18]. The DeepLab [4, 19] series enhances the accuracy of semantic segmentation by expanding the receptive field and contextual information.

### 2.3. Road Extraction

At present, road extraction is mainly through visual interpretation to obtain accurate results. In the past few years, the machine learning method represented by HOG [20] and SVM [21] has made some progress. eCognition [22] was the first object-oriented remote sensing interpretation software in the world. It proposes a revolutionary classification technology that provides new ideas for road extraction. The use of deep learning technology for remote sensing image interpretation is mainly divided into two categories, semantic segmentation [23] and edge detection [24]. In this paper, we use the method of semantic segmentation for road extraction.

More recent works [25] iteratively and hierarchically merge the feature hierarchy to better fuse information across layers. [26] Propose a novel object context polling (OCP) to aggregate the information according to the object context. [27] Propose global contextual information and selectively highlight class-dependent feature maps. [28] Propose exchange information among feature maps corresponding to adjacent scales. SENet [11] learns the importance of each channel automatically by learning and then according to this importance to enhance useful features and suppress features that are not useful for the current task. Self-supervised learning [29] and meta-learning [30] can also be applied in semantic segmentation. All of these methods are obtained a common semantic segmentation architecture by changing the network architecture or to propose a generic module. In contrast, our model is designed for the three urban areas of Yinchuan city and has certain specificity. By considering the local complex topographic features and the natural properties of the local roads, we add the ASPP module after the feature extraction based on the encoder-decoder architecture to further extract the multiscale context information of the image. By acquiring the multiscale context information of the image, the network can adapt the multiscale roads in different terrains, making it easy to classify pixels.

## 3. Our Approach

In this section, we introduce our proposed network architecture, then elaborate on the composition of each module, and then solve the problems. We also present a combined loss function which further improves performance by better optimizing mIoU.

### 3.1. Network Architecture

**Figure 1:**
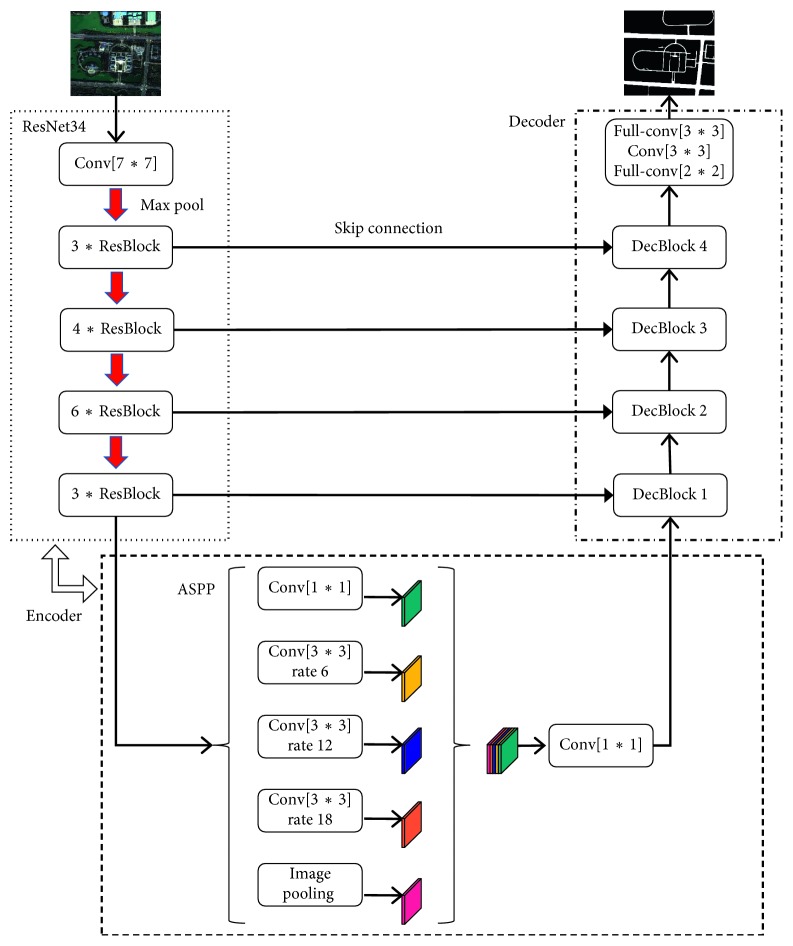
Our network architecture.

The network architecture is presented in Figure 1. It is divided into two parts: an encoder for extracting feature maps and a decoder for restoring feature map resolution. The encoder consists of ResNet34 and ASPP modules, and the decoder uses the same decoder as LinkNet. ResNet is proposed to solve the problem of performance degradation with the increase of network depth; the baseline architectures are the same as the plain nets, except inserting shortcut connections to constitute a residual network. ASPP adopts a structure similar to spatial pyramids pooling, whose purpose is to obtain more robust segmentation results with multiscale information. It uses four parallel atrous convolutions with different atrous rates and image-level features to obtain multiscale context information of images. The decoder uses the same decoder as LinkNet, which focuses on the speed of semantic segmentation. At the same time, the encoder mapping is connected to the decoder of the corresponding size, and the calculation speed is greatly improved under the premise of ensuring accuracy.

#### 3.1.1. Encoder

*ResNet34*. Semantic segmentation tasks usually require feature extraction through convolution operations to generate feature maps with lower resolution. In order to achieve higher precision, we began to deepen the number of layers in the network, but as the number of network layers increases, the gradient disappearance problem and the gradient explosion problem usually become more and more obvious, which leads to network convergence very slowly or even no convergence. Of course, deep convolutional neural networks can converge by adding regular terms and changing the optimized activation function. At this point, there will be a downgrade problem: as the network depth increases, the accuracy of the training set decreases. The downgrade problem indicates that deep networks are not well optimized. ResNet was officially proposed to address the downgrading problem, so we use ResNet34 as the primary feature extractor.

**Figure 2:**
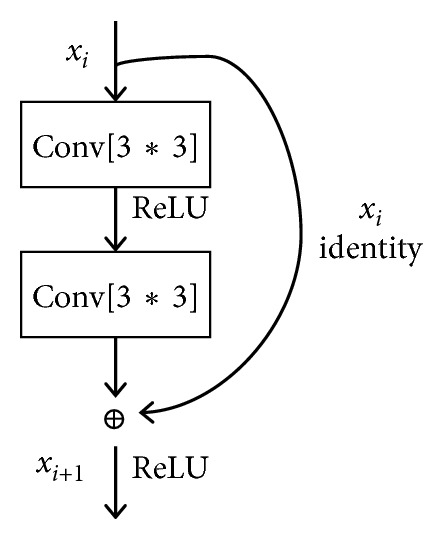
The residual block.

The basic composition of the residual network is the residual block (Figure 2), and each residual block is composed of two layers of convolution. Input an image *x*_*i*_, finish convolution and ReLU operation, and get the residual mapping defined as

(1) $$\begin{array}{c} F\left(x,\;\left\{W_{i}\right\}\right)=W_{2}\delta \left(W_{1}x_{i}\right), \end{array}$$

where *x*_*i*_ is the input vectors of the residual block, *δ* represents the ReLU operation, and *W*_*i*_ is a connection matrix to match the dimension. At the same time, the input image passed to a shortcut connection, and get identity mapping *W*_s_*x*_*i*_. Then, input the residual mapping and identity mapping to elementwise addition and get the output of the residual block defined as

(2) $$\begin{array}{c} x_{i+1}=F\left(x,\;\left\{W_{i}\right\}\right)+W_{\text{s}}x_{i}, \end{array}$$

where *W*_s_ is used to match the dimensions when the dimensions of *x*_*i*_ and *F* are different. Note that the function *F*(*x*, {*W*_*i*_}) can represent multiple convolutional layers, and the elementwise addition indicates that each of the two feature maps is performed on a channel-by-channel and element-by-element.

*ASPP*. Road extraction is a difficult task in Yinchuan city. The terrain is complex, and the roads are displayed in various forms; the construction standards of the road are different, such as different widths and different materials; the roads have serious occlusion problems, such as shadows and trees. In response to these problems, we connect ASPP as a key element of the network architecture after ResNet34, further extract multiscale context information from feature map, and then fuse the extracted information. Among ASPP, atrous convolution can solve the problem of resolution reduction caused by DCNNs while adjusting the receptive field of the filter and does not add new additional learning parameters; atrous rates represent the step size of sampling the input feature map, and modifying the value of atrous rate can change the receptive field of the filter and can also control the compactness of the feature of the full convolution network. In our network architecture, ASPP includes a 1 *∗* 1 convolution and three 3 *∗* 3 convolutions with atrous rates of 6, 12, and 18, and the image-level features (Figure 1).

#### 3.1.2. Decoder

After obtaining the feature map of the image by the encoder, we restore the feature map to the size of the original image by the decoder for dense prediction. Our expectation of intensive prediction is to ensure the speed without loss of accuracy. Through experimental comparison, LinkNet contains fewer parameters, the whole network is more efficient, and even real-time operation can be realized. In the encoder, the feature map is obtained by multiple convolution and downsampling operations. Some spatial information is lost during the downsampling process, and it is difficult to perfectly recover the lost information only by the upsampling operation of the decoder. Therefore, the encoder map is contacted to the corresponding decoder through the skip connection, and the decoder can directly obtain the information learned by the encoder, and the decoder utilizes the low-level accurate position information without adding additional parameters and calculations, thereby improved accuracy and speed.

**Figure 3:**
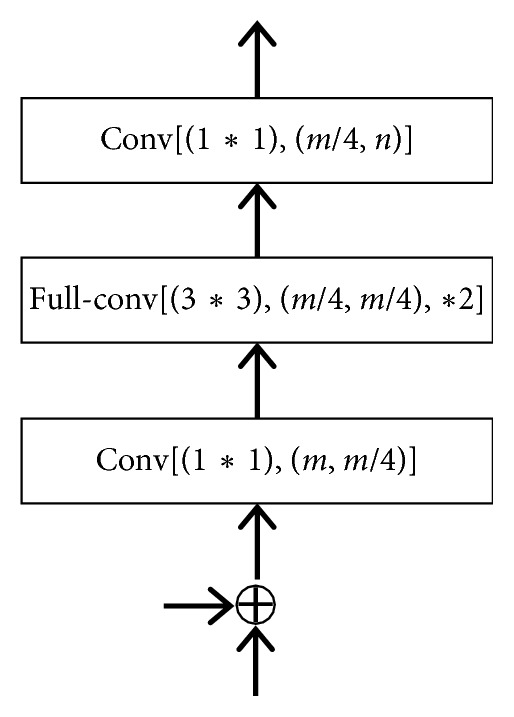
The decoder block.

The decoder in our network architecture consists of four decoder blocks, each consisting of two convolutions and one full convolution. The input of the decoder block consists of two parts: the output of the previous layer and the encoder map from skip connection (Figure 3). In addition, there is a batch normalization between each convolutional layer and which is followed by ReLU nonlinearity and *∗*2 means upsampling by a factor of 2.

### 3.2. Loss Function

Road extraction is a serious unbalanced task in which the road occupies a small part of the entire picture and the rest is the background. There are two ways to solve these problems: on the one hand, balancing the dataset; on the other hand, improving the loss function. We use the latter to improve model performance.

The common loss function for semantic segmentation is the pixel-level cross-entropy loss, which is checked for each pixel individually and compares the class prediction to the one-hot encoded target vector. Since the cross-entropy loss function separately evaluates the classification prediction of each pixel vector and then averages all the pixels, we can consider that the loss function treats each pixel in the image equally. However, our dataset category is heavily unbalanced, and the background is trained too many times, which leads to the training results tend to background. Another popular loss function in semantic segmentation is the loss function based on the Dice coefficient, which essentially measures the degree of overlap between two samples. The Dice coefficient calculation formula is as follows:

(3) $$\begin{array}{c} \text{Dice}=\frac{2\left|A\;\;\cap \;\;B\right|}{\left|A\right|+\left|B\right|}, \end{array}$$

where |*A* ∩ *B*| represents the number of common elements of the set *A* and *B*, |*A*| represents the number of elements of the set *A*, and |*B*| represents the number of elements of the set *B*. In equation (1), the numerator focuses on the intersection between the prediction and the target mask, and the denominator is related to the amount of activation in each mask. This produces an effect of normalizing the loss based on the size of the target mask. Thereby, those categories with fewer samples in the network learning image can be enhanced. We note that Berman et al. [31] proposed the Lovasz-Softmax loss to optimize the evaluation index IoU for the segmentation task. In order to improve the accuracy of our predictions, we combine binary cross-entropy (BCE), Dice coefficient, and Lovasz-Hinge loss as our final loss function. The final loss function is as follows:

(4) $$\begin{array}{c} L_{\text{f}}=\left(1\;\;-\;\;\alpha \right)\;*\;\left(L_{\text{BCE}}\;\;+\;\;L_{\text{DC}}\right)+\alpha \;*\;L_{\text{LH}}, \end{array}$$

where *L*_BCE_ represents the BCE loss, *L*_DC_ represents the loss function based on the Dice coefficient, *L*_LH_ represents the Lovasz-Hinge loss, and *α* controls the weight of different loss.

## 4. Experiments

This section provides a through the description of our experiments. We will start with the dataset and then explain our experimental details and evaluation criteria and finally show our experimental results.

### 4.1. Datasets

In our experiments, the satellite image used for road extraction comes from the Gaofen-2 satellite. It covers three districts of Yinchuan city: Xingqing District, Jinfeng District, and Xixia District, with a total area of 2,045 km^2^, and the ground resolution of the image pixels is 100 cm/pixel. Our goal is to extract the roads from the three districts. The labeled dataset consists of 304 images from the Gaofen-2 satellite and 6,226 images from [32], all of which are 1,024 *∗* 1,024 pixels in size and covered 1,950 km^2^ in total. The labeled dataset split into train/validation/test sets, each with 4,571/653/1,306 images (corresponding to a split of 70%/10%/20%). The three districts of Yinchuan city are used as application scenarios.

The data from [32] contain only rural and urban areas, and the terrain is relatively simple. The areas where we need to extract roads include not only rural areas and urban areas, but also mountainous, alluvial plains, deserts, photovoltaic power plant areas, and woodland areas. Different terrains have their own characteristics, and the types of roads are different, also the impact on road extraction is also different. The data from [32] cannot adapt to the complex terrain in the experimental area. The data from the Gaofen-2 satellite with labeled dataset are too little, so we use the external dataset [32] to expand the training data of our task.

To demonstrate the superiority of our proposed method, we validated our model on the Massachusetts Roads dataset [33]. The Massachusetts Roads dataset contains train/validation/test sets with 1,108/14/49 images, each with a size of 1,500 *∗* 1,500 pixels. We fill all the images to 1,536 *∗* 1,536 pixels and then crop each image into 512 *∗* 512 pixel images. In order to improve the generalization ability of the model, the Massachusetts Roads dataset has partially occluded part of the image. The cropped image has the entire image occluded, so we delete this part of the images in train sets. After a series of operations, the processed dataset of the Massachusetts Roads finally contains 8,616/126/441 images, corresponding to the train set/validation/test sets, and the size of each image is 512 *∗* 512 pixels.

### 4.2. Evaluation Criteria

In our experiments, we used pixel accuracy (PA) and Jaccard index as the evaluation criteria. PA refers to the ratio of the number of correctly classified pixels to the number of all pixels. Mean Intersection over union (mIoU) is the standard metric for semantic segmentation and refers to the intersection of the predicted segmentation and the ground truth divided by the union of the predicted segmentation and the ground truth.

Assume that TP is the number of pixels that are correctly predicted as road pixel, FP is the number of pixels that are wrongly predicted as road pixel, TN is the number of pixels that are correctly predicted as non-road pixel, and FN is the number of pixels that are wrongly predicted as non-road pixel for image. PA is expressed by the following equation:

(5) $$\begin{array}{c} \text{PA}=\frac{\text{TP}+\text{TN}}{\text{FP}+\text{FN}}. \end{array}$$

Assuming there are *n* images, the mIoU is defined as the average IoU among all images:

(6) $$\begin{array}{c} \text{mIoU}=\frac{1}{n}\overset{n}{\underset{i=1}{\sum }}{\text{IoU}}_{i}. \end{array}$$

### 4.3. Implementation Details

In the training phase, in order to avoid overfitting, we use some conventional means to augment the training dataset, including random Flip (horizontal, vertical, and diagonal), random Scale, random Shift, and Color Jittering. We trained the network for 100 epochs using the SGD optimizer and set the learning rate of each parameter group using a cosine annealing schedule, where a maximum number of iterations is 5, starting learning rate of 0.1 and decreasing it to 0.001. Weight decay and momentum values are set at 1e-4 and 0.9, respectively. *α* is set to 0.1 in the loss function.

In the test and application phase, in order to improve the robustness of our predictions, we implemented test time augmentation (TTA), which consists of 8 predicted averages, including horizontal flip, vertical flip, and diagonal flip.

### 4.4. Experimental Results

**Figure 4:**
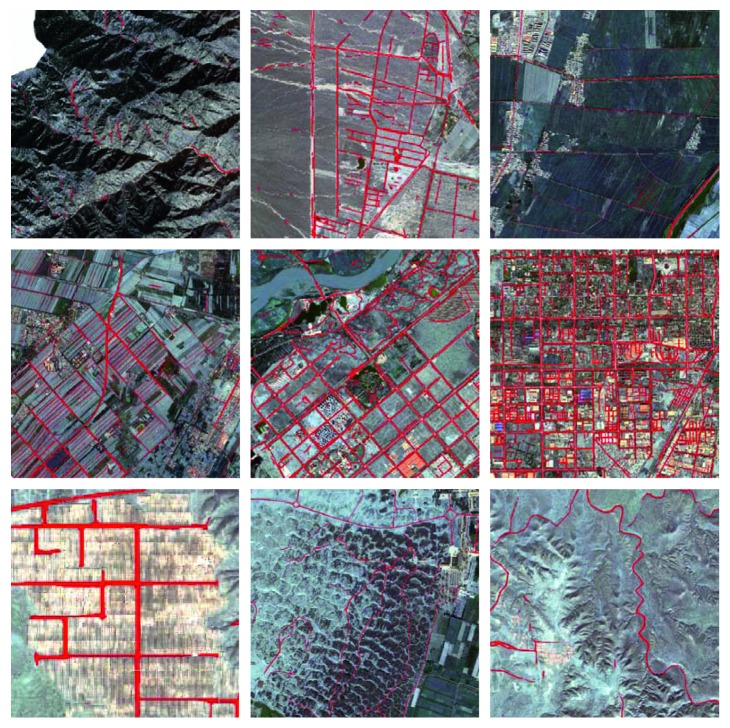
Example results of our method on road extraction in the Yinchuan city.

We randomly selected the prediction results of our model for road extraction under different terrains, as shown in Figure 4. As we have seen, our model can extract roads of different shapes in different terrains, such as rugged mountain roads, footpath in a field, and urban main roads.

As shown in Table 1, we compare our network architecture with the current model [3, 14, 19, 34]. Our model is 3.78% and 1.60% higher than the U-Net and LinkNet34 in the mIoU. This demonstrates the effectiveness of our proposed network architecture and loss function designed specifically for the Yinchuan city. At the same time, we noticed that our model has the same pixel accuracy as DeepLabv3+, which is down 0.8% on mIoU. There are two reasons for this. First, our model uses ResNet34 as the backend, and DeepLabv3+ uses Aligned Xception [19] as the backend. Second, the road label is not very accurate. Our model is slightly better than D-LinkNet on pixel accuracy and mIoU. The performance of pixel accuracy verifies that our ASPP module is slightly better than the center part of D-LinkNet. The performance of mIoU verifies that the loss function and the method of training the model are effective.

**Figure 5:**
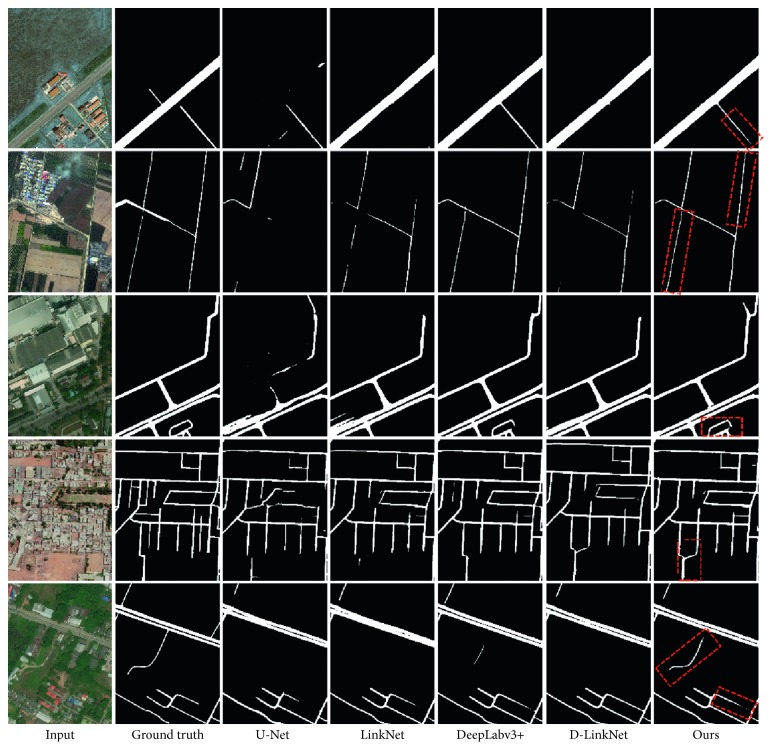
The extraction results of the U-Net model, LinkNet model, DeepLabv3+ model, D-LinkNet model, and our model. Note that our model can be adapted to multiscale road extraction.

Figure 5 shows the comparison of the specific extraction results. U-Net is weak and cannot adapt to the diversity characteristics of the road. For example, the main road in the first row of Figure 5 is not recognized. Secondly, U-Net performs poorly on detailed information and connectivity, such as the third, fourth, and fifth rows of Figure 5. The rest of the models have good road performance for most of the features. After comparison, we can find that our model is more complete in extracting the roads in the image, and the control of the details is more precise.

**Figure 6:**
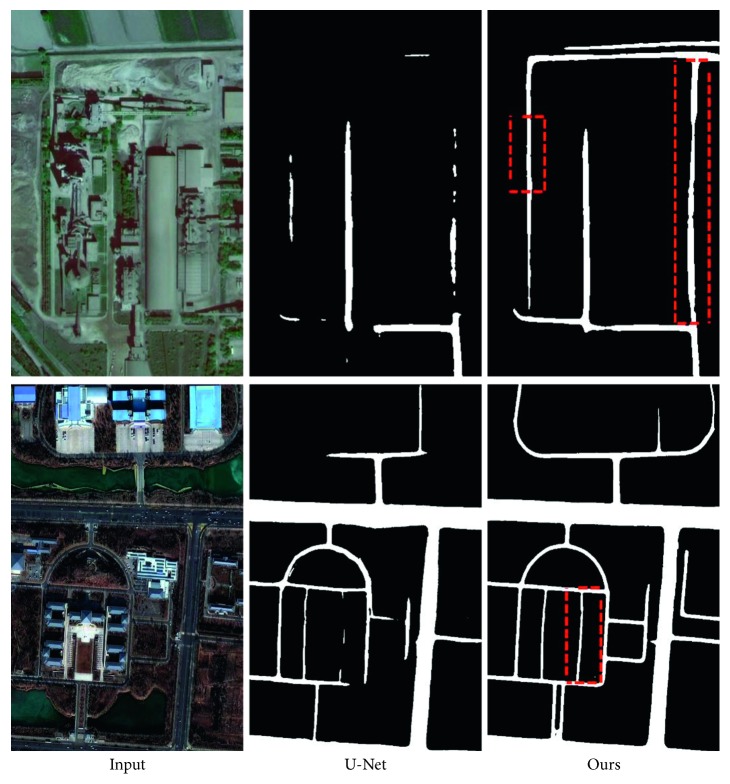
The extraction results of the U-Net model and our model. Note that our model can be adapted to multiscale road extraction.

Our method is robust to road connectivity problems caused by shadows, occlusions, etc. We compare the classic encoder-decoder model. As shown in the first row of Figure 6, our model can solve the road connectivity problem caused by flyover and shadow occlusion to some extent. In addition, our models are also tolerant of images of different color styles, as shown in the second row of Figure 6.

The experimental results on the Massachusetts Roads dataset are shown in Table 2. LinkNet performs best on pixel accuracy, and our model has the best performance on mIoU. The Massachusetts Roads dataset has the same road width (the road width is different in our dataset). The design of DeepLabv3+ and D-LinkNet mainly considers the multiscale concept of segmentation objects, although the atrous convolution increases the receptive field of the model, but the segmentation objects behave consistently. So these two models do not give full play to the advantages, but the network is simpler and LinkNet performance is slightly better. Our model also considers the concept of multiscale. At the same time, we have designed a new loss function for the optimization of mIoU, so it is reasonable for our model to perform best on mIoU.

## 5. Conclusions

In this paper, we presented a novel network for road extraction. We analyzed the special terrain of Yinchuan city and expanded our training datasets with external datasets. In terms of network architecture, we combine ASPP and classic encoder-decoder architecture and combine three loss functions to propose a loss function that is more suitable for our application scenario. Experiments show that our method can solve the road extraction task in Yinchuan city to a certain extent, and it has certain robustness to shadow, occlusion, and connectivity. This method has a good application prospect.

Currently, it is a very time-consuming and tedious task to manually label remote sensing images. The work in the paper is also a good practice to introduce external datasets. In the future, more attention will be taken on weakly supervised learning and meta-learning in order to improve the ability of our method.

## Figures and Tables

**Table 1:** Comparison with U-Net, LinkNet34, DeepLabv3+, and D-LinkNet on the test datasets.

Model	Pixel accuracy	mIoU
U-Net	0.978	0.793
LinkNet	0.981	0.810
DeepLabv3+	0.983	0.831
D-LinkNet	0.982	0.821
Ours	0.983	0.823

**Table 2:** Comparison with U-Net, LinkNet, DeepLabv3+, and D-LinkNet on the Massachusetts Roads test datasets.

Model	Pixel accuracy	mIoU
U-Net	0.9566	0.5985
LinkNet	0.9678	0.6792
DeepLabv3+	0.9667	0.6672
D-LinkNet	0.9675	0.6703
Ours	0.9672	0.6878

## Data Availability

The data used to support the findings of this study are available from the corresponding author upon request.
